# Comparison of hospitalized patients with severe pneumonia caused by COVID-19 and influenza A (H7N9 and H1N1): A retrospective study from a designated hospital

**DOI:** 10.1515/med-2022-0610

**Published:** 2022-12-09

**Authors:** Binbin Gu, Lin Yao, Xinyun Zhu, Peijun Tang, Cheng Chen

**Affiliations:** Department of Intensive Care Unit, Soochow University Affiliated Infectious Disease Hospital: The Fifth People’s Hospital of Suzhou, Suzhou, Jiangsu 215000, China; Department of Respiratory and Critical Medicine, The First Affiliated Hospital of Soochow University, Suzhou, Jiangsu 215000, China; Department of Respiratory and Critical Medicine, The First Affiliated Hospital of Soochow University, 899 Pinghai Road, Suzhou, Jiangsu 215000, China; Department of Pulmonary, Soochow University Affiliated Infectious Disease Hospital: The Fifth People’s Hospital of Suzhou, Suzhou, Jiangsu 215000, China; Department of Pulmonary, Soochow University Affiliated Infectious Disease Hospital: The Fifth People’s Hospital of Suzhou, 10 Guangqian Road, Suzhou, Jiangsu 215000, China

**Keywords:** pneumonia, influenza A, COVID-19

## Abstract

Considerable attention has been focused on the clinical features of coronavirus disease 2019 (COVID-19), but it is also important for clinicians to differentiate it from influenza virus infections. In the present study, the rate of coexisting disease was lower in the severe COVID-19 group than in the influenza A group (*p* = 0.003). Radiologically, severe COVID-19 patients had fewer instances of pleural effusion (*p* < 0.001). Clinically, severe COVID-19 patients had relatively better disease severity scores, less secondary bacterial infections, shorter times to beginning absorption on computed tomography, but longer durations of viral shedding from the time of admission (*p* < 0.05). Although the more severe influenza A patients required noninvasive respiratory support, these two groups ultimately yielded comparable mortalities. Based on the multiple logistic regression analysis, severe COVID-19 infection was associated with a lower risk of severe acute respiratory distress syndrome [odds ratio (OR) 1.016, 95% [confidence interval (CI)] 1.001–1.032, *p* = 0.041] and a better pneumonia severity index (OR 0.945, 95% [CI] 0.905−0.986, *p* = 0.009); however, these patients exhibited longer durations of viral shedding (OR 1.192, 95% [CI] 1.047−1.357, *p* = 0.008) than patients with severe influenza A infection. In conclusion, the conditions of severe influenza A patients appeared to be more critical than that of severe COVID-19 patients. However, relatively lower mortalities of these two severe cases are expected in the context of sufficient medical supplies.

## 1 Introduction

The 2019 novel coronavirus (SARS-CoV-2) and influenza A viruses are both viruses that primarily target the human respiratory system. Diseases associated with their infections vary from mild respiratory illness to acute pneumonia and even acute respiratory distress syndrome (ARDS) [1,2]. Currently, the Omicron variant of SARS-CoV-2 is responsible for the COVID-19 outbreak, and some study has provided the data of different patterns of clinic characteristics and reduced severity from infections that occurred in Omicron variant as compared with the ancestral variant [3,4]. Due to the vaccinations exist for COVID-19, these are still deemed to have limited understanding clinical comparison between severe influenza that causes hospitalization and severe COVID-19 cases whom being unvaccinated.

Some findings point to the activation of fundamentally different innate immune pathways in SARS-CoV-2 and influenza infection [5]. In addition, in all hospitalized patients, COVID-19 was reported with a higher number of complications and higher in-hospital mortality compared to influenza, even in a population with fewer comorbidities [6,7,8]. Comparatively, COVID-19 infections kept sweeping across the world at the same time, suggestive of its much higher infectivity than influenza. Furthermore, simultaneous or sequential coinfection by SARS-CoV-2 and A (H1N1) pdm09 caused more severe disease than monoinfection by either virus in hamsters [9].

Owing to the virus origin, it is important for clinicians to accurately identify these two respiratory viral infections under timely and proper treatment. In this study, we systematically compare severe pneumonia patients infected with ancestral variant virus of SARS-CoV-2 versus the influenza A virus in terms of clinical presentations, laboratory tests, virologic shedding, image characteristics, complications, and clinical outcomes in the context of critical bundle and intensive management to provide guidance for their differential consideration.

## 2 Materials and methods

### 2.1 Patients

All of the severe COVID-19 pneumonia (*n* = 27) and severe influenza A-induced pneumonia subjects (*n* = 43) were confirmed using laboratory tests and were hospitalized at the Fifth People’s Hospital of Suzhou. Influenza A, which comprises the highly pathogenic avian influenza H7N9 and human influenza H1N1, was included in this study. The severe COVID-19 pneumonia cases were hospitalized from January 2020 to March 2020, and these cases were defined according to the diagnostic and treatment guidelines for COVID-19 pneumonia issued by the National Health and Family Planning Commission of P.R. China (Version 1-8). The severe influenza A cases were hospitalized from 2014 to 2016, and these cases met the criteria published by the National Health and Family Planning Commission of P.R. China (the 2nd edition, 2013).

### 2.2 Data collection

The patient data were collected using an electronic case report form, and it included the following: demographic characteristics (age and sex), comorbidities, clinical symptoms, laboratory tests (blood routine test, arterial blood gas analysis, and blood chemistry), virologic test, microbiological findings, and images of the lung [chest computed tomography (CT)]. Antimicrobiological therapy, respiratory support, complications, and outcomes were also recorded. The Ethics Committee of the Fifth People’s Hospital of Suzhou approved this study (2020-005).

### 2.3 Study design

This was a retrospective case–control study. We compared two independent cohorts of patients infected with either COVID-19 or influenza A in terms of the initial onset, further course, and outcomes. These included the clinical presentations at diagnosis, virologic shedding, peak values of the laboratory tests, time courses of the image characteristics, the worst sequential organ failure assessment (SOFA) and pneumonia severity index (PSI) scores, extrapulmonary complications, secondary bacterial infections, intensive therapies, and clinical outcomes.

### 2.4 Definitions

Septic shock was defined according to the 2016 Third International Consensus Definition for Sepsis and Septic Shock [10]. Secondary bacterial infection was diagnosed when patients showed clinical symptoms or signs of pneumonia or bacteremia, and a positive culture of a new pathogen was obtained from lower respiratory tract specimens (qualified sputum, endotracheal aspirate, or bronchoalveolar lavage fluid) or blood samples after admission [11]. Extrapulmonary complication was defined as the following: (1) acute kidney injury was diagnosed according to the KDIGO clinical practice guidelines; (2) acute cardiac injury was diagnosed if the serum levels of the cardiac biomarkers (e.g., high-sensitive cardiac troponin I) were greater than the 99th percentile upper reference limit; (3) acute liver injury was diagnosed if the serum levels of alanine transaminase (ALT) or total bilirubin (TBIL) were greater than the two-fold of the upper reference limit; and (4) coagulopathy was defined as the three-second extension of the prothrombin time (PT) [12,13]. The RNA shedding duration was defined as the interval from admission to the date of the first RNA negative result prior to discharge.

### 2.5 Statistical analyses

Data are described as the mean ± standard deviation (SD), median (interquartile range), or number (%). Comparisons of the features between the different subtypes of virus (influenza A and COVID-19) were performed using a *t* test to compare the mean ± SD of the continuous variables. A Mann–Whitney *U* test was used to compare the medians of the continuous variables, and the Fisher exact test or the Chi-squared test was used to compare the proportions. To identify the risk factors associated with severe COVID-19 infection, we performed a multivariable logistic regression analysis adjusted for the baseline covariates. Statistical analyses were performed using SPSS version 24.0 for Windows, the probabilities were two-tailed, and a two-tailed *p* value of < 0.05 was considered significant.

## 3 Results

### 3.1 Demographic features

As shown in Table 1, the median age of the severe COVID-19 patients was 49 years old, which was comparable to that of the severe influenza A patients (60 years old, *p* > 0.05). The proportion of males in the severe COVID-19 patients was 74.07%, which was also comparable to that of the severe influenza A patients (69.77%, *p* > 0.05). A total of 25.93% of the severe COVID-19 patients had a history of underlying diseases, whereas that of the severe influenza A patients was significantly higher at 62.79% (*p* = 0.003). The majority of the severe influenza A patients suffered from hypertension (*p* < 0.001). There was no significant difference in the histories of diabetes or chronic-airway diseases between the two groups (*p* > 0.05).

**Table 1:** Characteristics of subjects

	Influenza A	COVID-19	*p* value
Age (median, years)	60.0 (41.0–71.0)	49.0 (38.0–59.0)	0.052
Male (%)	30 (69.77)	20 (74.07)	0.698
Underlying disease (%)	27 (62.79)	7 (25.93)	**0.003**
Hypertension	22 (51.16)	2 (7.41)	**<0.001**
HBV	2 (4.7)	0 (0)	0.519
Diabetes	5 (11.63)	4 (14.81)	0.983
Chronic-airway diseases	1 (2.33)	0 (0)	1.000
**Clinical manifestations (%)**			
Fever	42 (97.67)	26 (96.30)	1.000
Cough	42 (97.67)	21 (77.78)	**0.022**
Dyspnea	37 (86.05)	9 (33.33)	**<0.001**
Myalgia	12 (27.91)	5 (18.52)	0.373
Nasal congestion	10 (23.26)	1 (3.70)	0.064
Pharyngodynia	8 (18.60)	5 (18.52)	0.993
Gastrointestinal symptoms	5 (11.63)	3 (11.11)	1.000
Hemoptysis	2 (4.7)	1 (3.70)	1.000

The bold values have statistically significance (*p* < 0.05).

### 3.2 Clinical manifestations at diagnosis

Upon admission (Table 1), nearly all of the severe influenza A and severe COVID-19 patients presented with fever (97.67% vs 96.30%, *p* > 0.05). Furthermore, 97.67 and 86.05% of the severe influenza A patients had cough and dyspnea, and this was significantly greater than those of the severe COVID-19 patients (77.78, 33.33%, *p* < 0.05 for each). The proportions of myalgia (27.91%), nasal congestion (23.26%), pharyngodynia (18.60%), and gastrointestinal symptoms (11.63%) in the severe influenza A patients were comparable to those of the severe COVID-19 patients (18.52, 3.70, 18.52, and 11.11%), whereas hemoptysis was less common in both groups.

### 3.3 Laboratory results

Over the course of the hospitalization (Table 2), acute kidney injury and acute liver injury occurred in 11.63 and 62.79%, respectively, of the patients with severe influenza A, and this was comparable to those of the severe COVID-19 patients (11.11 and 77.78%, respectively, *p* > 0.05 for each). However, 37.21% of the severe influenza A patients suffered acute cardiac injury, which was significantly higher than the proportion of 3.70% in the severe COVID-19 patients (*p* = 0.001). Following biochemical testing, the peak levels of the TBIL, creatinine (Cr), and troponin I (TnI) in the severe COVID-19 patients were comparable to those in the severe influenza A patients (20.54 ± 2.51 vs 24.45 ± 3.02 mmol/L, 79.68 ± 4.61 vs 83.29 ± 9.40 μmol/L, and 17.22 ± 7.99 vs 160.52 ± 100.52 pg/mL, respectively, *p* > 0.05 for each).

**Table 2:** Over disease’s course between of severe influenza A and COVID-19 patients

	Influenza A	COVID-19	*p* value
Onset to confirm diagnosis (days)	7.40 ± 0.425	8.56 ± 0.69	0.134
Severity^*^			
Sepsis shock (%)	3 (6.98)	0 (0)	0.426
SOFA score	5.12 ± 0.53	3.44 ± 0.27	**0.007**
PSI score	89.51 ± 5.28	61.22 ± 2.68	**<0.001**
OI	147.32 ± 9.02	218.32 ± 11.26	**<0.001**
**Complication (%)**			
Lymphopenia	38 (88.37)	22 (81.48)	0.652
Thrombocytopenia	13 (30.23)	3 (11.11)	0.064
Acute kidney injury	5 (11.63)	3 (11.11)	1
Acute liver injury	27 (62.79)	21 (77.78)	0.189
Acute cardiac injury	16 (37.21)	1 (3.70)	**0.001**
Coagulation	22 (51.16)	4 (14.81)	**0.002**
Laboratory test^*^			
Ly (×10^9^/mL)	0.57 ± 0.06	0.73 ± 0.06	0.050
PLT (×10^9^/mL)	150.30 ± 12.28	161.89 ± 10.08	0.509
ALT (U/L)	105.40 ± 12.40	149.22 ± 19.11	**0.048**
TBIL (mmol/L)	24.45 ± 3.02	20.54 ± 2.51	0.368
TnT (pg/mL)	160.52 ± 100.52	17.22 ± 7.99	0.259
D-dimer (μg/mL)	5529.47 ± 898.24	1930.00 ± 363.30	**<0.001**
PT (s)	18.18 ± 1.68	13.10 ± 0.34	**0.005**
Cr (μmol/L)	83.29 ± 9.40	79.68 ± 4.61	0.773
**Positive bacterial culture (%)**			
Gram-pos	13 (30.23)	1 (3.70)	**0.007**
Gram-neg	5 (11.63)	2 (7.41)	0.870
**Radiologic findings**			
Ground-glass opacity	23 (53.49)	12 (44.44)	0.461
Crazy-paving pattern	4 (9.30)	8 (29.63)	0.061
Consolidative	37 (86.05)	25 (92.59)	0.651
Pleural effusion	29 (67.44)	4 (14.81)	**<0.001**

The bold values have statistically significance (*p* < 0.05).

^*^Minimal or peak value of the index over disease’s course.

Both the severe COVID-19 and the severe influenza A patients exhibited impairments in coagulation. However, significantly increased peak levels of D-dimer and PT were associated with the severe influenza A patients compared to the severe COVID-19 patients (5529.47 ± 898.24 vs 1930.00 ± 363.30 mg/L and 18.18 ± 1.68 vs 13.10 ± 0.34 s, *p* < 0.05 for each).

In terms of blood cell counts, lymphopenia was observed in the most severe COVID-19 (81.48%) and severe influenza A patients (88.37%, *p* > 0.05). Thrombocytopenia was observed in 11.11% of the severe COVID-19 and 30.23% of the severe influenza A patients (*p* > 0.05). The minimal level of platelets in the severe COVID-19 patients was comparable to those in the severe influenza A patients (161.89 ± 10.08 vs 150.30 ± 12.28 × 10^9^/L, respectively, *p* > 0.05).

The worst oxygenation index (OI) during hospitalization predicts deteriorated respiratory failure. The minimal level of OI in the severe COVID-19 patients was 218.32 ± 11.26 mmHg, which was significantly higher than the 147.32 ± 9.02 mmHg of the severe influenza A patients (*p* < 0.001).

### 3.4 Imaging findings

**Figure 1:**
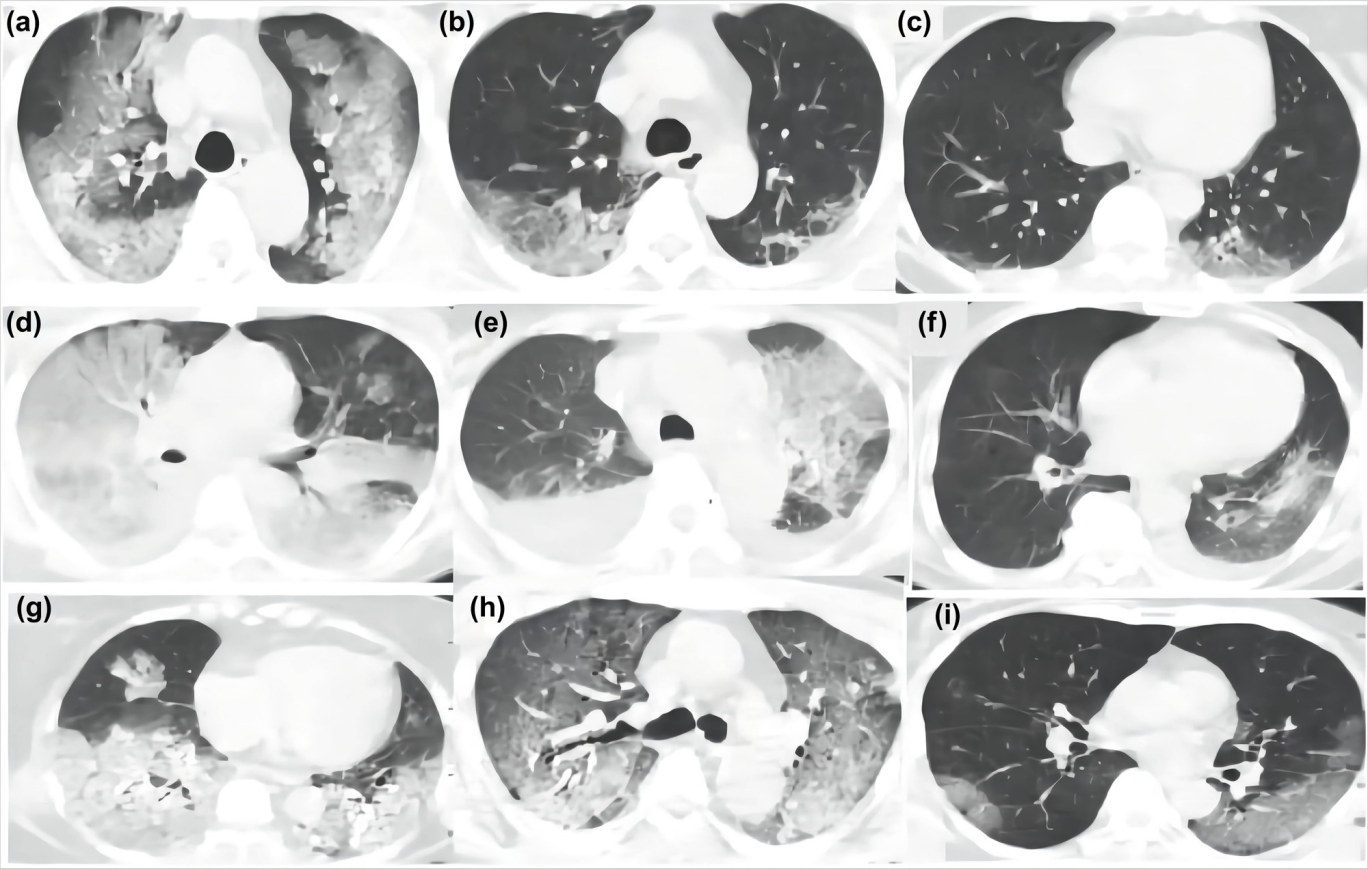
Chest CT images of patients with severe COVID-19 (a–c), H7N9 (d–f), and H1N1 (g–i). Ground-glass opacity was common in both the COVID-19 group and the influenza A group. Pleural effusion was more common in the influenza A group, and the crazy-paving pattern was more common in the COVID-19 group.

In terms of imaging characteristics (Figure 1, Table 2), consolidation and the ground-glass opacity (GGO) in the initial chest CTs were common in the COVID-19 patients and in the influenza A patients (92.59 vs 86.05% and 44.44 vs 53.49%, respectively, *p* > 0.05 for each). In contrast, patients with COVID-19 tended to have crazy-paving patterns compared to those with influenza A (*p* = 0.061). Furthermore, pleural effusion was more common in the influenza A patients than in the COVID-19 patients (*p* < 0.001).

### 3.5 Further course and intensive treatment

Over the course of the viral infections (Table 2), septic shock occurred in 6.98% of patients with severe influenza A. There was no significant difference in the duration of onset to ARDS between the severe influenza A and the severe COVID-19 patients (7.40 ± 0.43 and 8.56 ± 0.69 days, respectively *p* = 0.134). The highest SOFA score and PSI score of the severe COVID-19 patients were 3.44 ± 0.27 and 61.22 ± 2.68, respectively, which were lower than the scores of 5.12 ± 0.53 (*p* = 0.007) and 89.51 ± 5.28 (*p* < 0.001), respectively, for the severe influenza A patients.

All patients received a medical bundle intervention that included antimicrobial therapy, fluid administration, respiratory support, or steroid therapy.

The two groups presented with a variety of accompanying secondary bacterial infections. A total of 30.23% of the severe influenza A patients had positive cultures of pathogens isolated from qualified lower respiratory tract specimens, whereas that of the severe COVID-19 patients was significantly lower (3.70%, *p* = 0.007).

In terms of respiratory support (Table 3), during the entire process of treatment, the proportions of severe influenza A patients who received noninvasive mechanical ventilation (NIV) was significantly higher than that of the COVID-19 patients (58.14 vs 2.63%, respectively, *p* = 0.02). The failure rates of NIV in the severe influenza A patients were comparable to than those in the COVID-19 patients (4.65 vs 0%, respectively, *p* > 0.05).

**Table 3:** Treatment and prognosis of two groups

	Influenza A	COVID-19	*p* value
**Respiratory support (%)**			
NPPV	25 (58.14)	8 (29.63)	**0.020**
IMV	6 (13.95)	0 (0)	0.112
**Respiratory support failure (%)**			
NPPV	2 (4.65)	0 (0)	0.519
IMV	3 (6.98)	0 (0)	0.426
CRRT (%)	3 (6.98)	0 (0)	0.426
Duration of virus shedding (days)	10.95 ± 0.65	14.37 ± 1.41	**0.034**
The time to beginning absorption on CT (days)	13.31 ± 0.76	9.56 ± 0.52	**<0.001**
Glucocorticoid (%)	33 (76.74)	22 (81.48)	0.638
LMWH (%)	19 (44.19)	14 (51.85)	0.532
Mortality (%)	3 (6.98)	0 (0)	0.426

The bold values have statistically significance (*p* < 0.05).

In addition to the treatments above, 81.48% of COVID-19 patients received glucocorticoids, and this was comparable to the proportion of 76.74% in the influenza A patients (*p* > 0.05). Low-molecular-weight heparin (LMWH) was administered in 51.85% of the COVID-19 patients, which also was comparable to that administered to the influenza A patients (44.19%, *p* > 0.05).

### 3.6 Virologic outcomes and prognosis

All of the patients received antiviral therapies. Oseltamivir was administered in all of the influenza A patients. However, the COVID-19 patients had a variety of antiviral treatments, including 77.78% with lopinavir/ritonavir, 55.56% with arbidol, and 40.74% with a combination. The duration of the severe COVID-19 RNA shedding upon admission was 14.37 ± 1.41 days, which was longer than that of the severe influenza A patients (10.95 ± 0.65 days, *p* = 0.034).

Based on the follow-up of the chest CT, the time to beginning absorption on chest CT (TTBAC) was established. The severe influenza A patients had longer TTBACs than those in the severe COVID-19 patients (13.31 ± 0.76 vs 9.56 ± 0.52 days, respectively, *p* < 0.001).

In terms of prognosis, although the in-hospital mortality of the influenza A patients with ARDS was 6.98%, it did not reach statistical significance when compared to the COVID-19 patients (0, *p* = 0.426).

### 3.7 Multivariate analysis

Based on the multiple logistic regression analysis, compared with the parameters in the severe influenza A patients, the severe COVID-19 patients were associated with a lower risk of the presence of severe ARDS (odds ratio (OR) 1.016, 95% [confidence interval (CI)] 1.001−1.032, *p* = 0.041) and a better PSI score (OR 0.945, 95% [CI] 0.905−0.986, *p* = 0.009) but exhibited a longer duration of viral shedding (OR 1.192, 95% [CI] 1.047−1.357, *p* = 0.008) than that of the severe influenza A infections (Table 4).

**Table 4:** Multivariate analysis of independent risk factors for differentiating COVID-19 from influenza A infection

Variable	Univariate analysis, OR (95% CI)	*p* value	Multivariate analysis, OR (95% CI)	*p* value
Age (years)	0.968 (0.937–1.000)	0.051		
Male	1.238 (0.421–3.642)	0.698		
Duration of virus shedding (days)	1.116 (1.015–1.226)	**0.023**	1.192 (1.047–1.357)	**0.009**
SOFA score	0.745 (0.564–0.983)	**0.038**	1.378 (0.781–2.430)	0.268
PSI score	0.944 (0.913–0.976)	**0.001**	0.945 (0.905–0.986)	**0.041**
OI	1.019 (1.009–1.029)	**<0.001**	1.016 (1.001–1.032)	0.086
Ly (×10^9^/mL)	4.099 (0.946–17.767)	0.059		
PLT (×10^9^/mL)	1.002 (0.996–1.009)	0.505		
ALT (U/L)	1.006 (1.000–1.011)	0.059		
TBIL (mmol/L)	0.985 (0.92–1.019)	0.377		
D-dimer (μg/mL)	1.000 (0.999–1.000)	**0.014**	1.000 (0.999–1.000)	**0.008**
TnT (ng/mL)	0.987 (0.971–1.002)	0.085		
Cr (μmol/L)	0.998 (0.988–1.009)	0.771		

The bold values have statistically significance (*p* < 0.05).

## 4 Discussion

In this study, we compared the clinical features and courses of patients with severe pneumonia caused by COVID-19 and influenza A. As sufficient medical staff and medical supplies have affected the treatments and prognoses of severe cases, this retrospective study was conducted in a resident designated hospital where medical resources reached standardized respiratory support in accordance with the related guidelines.

Because of the different therapies, it is important to differentiate these two diseases using the clinical presentations. We found that compared with the features of influenza A patients, the COVID-19 patients were less inclined to exhibit cough and dyspnea. Therefore, we speculate from previous research that the severe influenza A infection may present as more respiratory symptoms compared with the COVID-19 infection. Furthermore, based on the proportions of underlying diseases in these two groups, it was indicated that the combination of underlying diseases had a significant effect on the severities of the influenza A infections [14,15,16,17,18].

The primary manifestations on the chest CT for both diseases were characterized by the consolidative and GGOs. In addition, the severe COVID-19 patients tended to have crazy-paving pattern imaging, whereas pleural effusions were more frequent in the severe influenza A patients. It is suggested that a combination of the radiologic findings might have a certain value in the differential diagnosis of the two diseases.

Clinically, although severe COVID-19 patients had similar extrapulmonary complications to those observed in the severe influenza A patients, they were less inclined to suffer from secondary bacterial infections, acute cardiac injuries, and impairments in coagulation. The majority of patients had increased coagulation activities marked by increased D-dimer concentrations. High levels of D-dimer have a reported association with the 28-day mortality in patients with infection or sepsis [19]. These experimental indexes were considered to be closely related to the severities of the severe infections [13,20].

In terms of disease severity, the duration from the onset to a confirmed diagnosis of ARDS in the severe influenza A and COVID-19 patients was comparable. However, the severe COVID-19 patients were more inclined to have a relatively better disease severity score. In addition, according to the PaO_2_/FiO_2_ in the severe COVID-19 patients, the corresponding OI was significantly higher than that of the severe influenza A patients, suggesting relatively moderate conditions of the severe COVID-19 patients. The serial chest CT showed that the absorption of the lesions among the influenza A patients was slow. The primary cause may have been more severe damage to the lung tissue.

Although some therapies and vaccines have received FDA approval or emergency use authorization, the rapid spread of the virus still poses a global health emergency [21]. Under the circumstances, a comparison of the duration of virus shedding between the severe COVID-19 and influenza A infections may produce more interesting findings. Surprisingly, the severe COVID-19 group had a longer duration of viral shedding upon admission than the severe influenza A group. This has important implications for guidance regarding the length of antiviral treatment.

We also found that severe COVID-19 patients received a wider variety of treatments that were similar to severe influenza A patients. The application of glucocorticoids and LMWH were both common in the COVID-19 and influenza A patients in this study. The treatments for ARDS of the two groups were primarily based on noninvasive positive pressure ventilation (NPPV). More patients with severe influenza A required NPPV and were ultimately cured. However, additional prospective and comparative trials that address the need for intubation and mortality rates are required [22,23].

However, there were some limitations of this study. First, this was a retrospective study that included data from one independent single-center cohort, and this may have resulted in unavoidable bias. Second, more observations are required to further clarify the clinical features using a large-scale investigation.

## 5 Conclusion

This study was to help understand the similarities and dissimilarities between unvaccinated patients with influenza A and ancestral variant of SARS-CoV-2 complicated by severe pneumonia. Even no specific anti-virus treatment, given sufficient medical staff and medical supplies, the relatively lower mortality rates of these two severe cases were to be expected. However, when facing the flu season amid the COVID-19 pandemic, vaccinations of both flu and COVID-19 should be reinforced, along with close monitoring of COVID-19-positive population coinfected with influenza A.

## Abbreviations

ARDS: acute respiratory distress syndrome
CI: confidence interval
COVID-19: Coronavirus Disease 2019
CT: computed tomography
GGOs: ground-glass opacities
NPPV: noninvasive positive pressure ventilation
OR: odds ratio
PSI: pneumonia severity index
SOFA: sequential organ failure assessment
